# Long-Term Changes in the Diet of *Gymnogobius isaza* from Lake Biwa, Japan: Effects of Body Size and Environmental Prey Availability

**DOI:** 10.1371/journal.pone.0053167

**Published:** 2012-12-28

**Authors:** Jonathan Carlo Briones, Cheng-Han Tsai, Takefumi Nakazawa, Yoichiro Sakai, Rey Donne S. Papa, Chih-hao Hsieh, Noboru Okuda

**Affiliations:** 1 Graduate School, University of Santo Tomas, Manila, Philippines; 2 Institute of Oceanography, National Taiwan University, Taipei, Taiwan; 3 Institute of Ecology and Evolutionary Biology, National Taiwan University, Taipei, Taiwan; 4 Center for Ecological Research, Kyoto University, Otsu, Japan; 5 Department of Biological Sciences and Research Center for the Natural and Applied Sciences, University of Santo Tomas, Manila, Philippines; Aristotle University of Thessaloniki, Greece

## Abstract

Body size and environmental prey availability are both key factors determining feeding habits of gape-limited fish predators. However, our understanding of their interactive or relative effects is still limited. In this study, we performed quantitative dietary analysis of different body sizes of goby (*Gymnogobius isaza*) specimens collected from Lake Biwa between 1962 and 2004. First, we report that the diet was composed mainly of zooplankton (cladocerans and copepods) before the 1980s, and thereafter, shifted to zoobenthos (gammarids). This foraging shift coincided with, and thus can be linked to, known historical events in the lake at that time: decrease in zooplankton abundance with the alleviation of eutrophication, increase in fish body size resulting from fish population collapse, and increase in gammarid abundance due to reduced fish predation pressure. Supporting this view, our data analyses revealed how the long-term changes in the diet composition would be co-mediated by changes in fish body size and environmental prey availability. Specifically, while zoobenthos abundance strongly affected the fish diet composition, larger (smaller) fish preferred zoobenthos (zooplankton). Furthermore, the body size effects were stronger than those of prey availability. These results provide the best long-term evidence that fish feeding habits vary over decades with its body size and prey community due to anthropogenic disturbances.

## Introduction

Fish predation plays a key role in controlling structure and dynamics of aquatic food webs [1], [2], [3]. Dietary studies using stomach content analysis have long been used to reveal aspects of fish feeding habits and to understand aquatic food webs [4], [5], [6], [7], [8]. For example, investigation of the relationship between fish diets and environmental prey availability or system productivity has helped characterize bottom-up processes regulating energy flows from lower to higher trophic levels [9], [10], [11]. In addition, dietary analyses also indicate how fish select food items among diverse types of potential prey [12], [13]. In particular, fish often exhibit marked ontogenetic changes in diet, which is typically caused by gape-limited feeding [14], [15]. As such, fish can have flexible (i.e., temporally variable) feeding habits depending on their body size and prey availability. Scrutinizing such trophic flexibility and identifying its abiotic and biotic determinants are crucial for understanding aquatic food webs and for ultimately predicting ecosystem responses to environmental changes due to anthropogenic disturbances.

Although what fish eat and what determines it have long been central subjects of aquatic ecology [2], [5], [6], our understanding of the fish feeding habits is still limited. Many researchers have reported temporal changes in fish feeding habits in both freshwater and marine systems, and most of these studies have shown that fish feeding habits are highly associated with environmental prey availability (e.g., [10], [16], [17]) or fish body size (e.g., [18], [19], [20]). In general, however, the effects of prey availability have been tested with long-term data without accounting for intraspecific fish body size variations [10], [16], [17], while the effects of fish body size have been tested with short-term or snapshot data focusing on ontogenetic diet shifts without considering the possible effects of environmental prey variability [18], [19], [20]. Therefore, to date, long-term stomach content data analyzed in relation to changes in both fish body size and environmental prey availability have been very limited, and their interactive or relative effects on fish feeding habits are still poorly understood. This is especially true and important for freshwater fish, considering the facts that there is a research bias towards marine species in fish feeding ecology [21] and that freshwater ecosystems are highly vulnerable to anthropogenic disturbances [22], [23].

**Figure 1:**
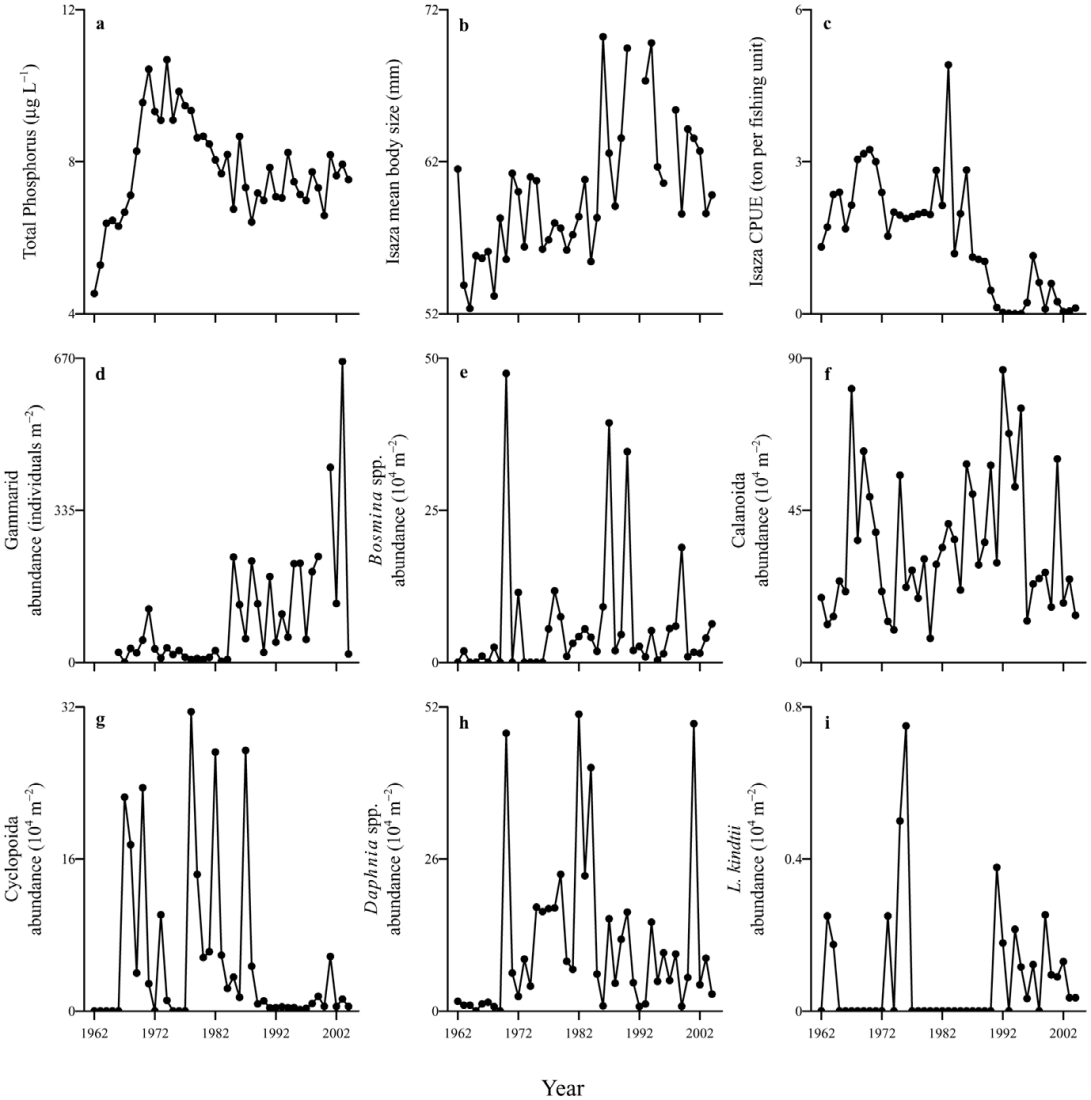
Time-series data in Lake Biwa. (a) the annual average total phosphorus concentration in the upper 20 m of the water column, (b) the average total body length and (c) fishery catch per unit effort of *Gymnogobius isaza*, and the annual average abundances of (d) gammarids, (e) *Bosmina* spp., (f) Calanoida, (g) Cyclopoida, (h) *Daphnia* spp., and (i) *Leptodora kindtii*.

In the present study, we examine long-term (spanning more than 40 years) changes in the diet composition of *Gymnogobius isaza* (Tanaka 1916) by performing extensive and quantitative dietary analyses using archival fish specimens that have been collected from Lake Biwa, Japan for over four decades (1962–2004). Lake Biwa (35°20′N 136°10′E) is the largest lake in Japan with an area of 670 km^2^ and a maximum depth of 104 m. Organisms living in Lake Biwa have been subject to various ecosystem disturbances during the latter half of the 20th century. The lake was originally oligotrophic but became eutrophic in the 1960s because of increased nutrient loading (Fig. 1a; data from [24]). Regulations on nutrient discharge into the lake were enforced by the local government in 1980, after which eutrophication became progressively alleviated, and the lake finally stabilized in a mesotrophic state after the mid-1980s [24]. During the period of eutrophication, zooplankton, one of the main prey of *G. isaza* [25], increased and maintained elevated levels of abundance during the 1970s, and thereafter, their abundance progressively decreased (Figs. 1e–i; data from [26]; also see [27]). Another significant disturbance was the invasion of exotic predatory fish, such as bluegill (*Lepomis macrochirus*) and largemouth bass (*Micropterus salmoides*), in the 1960s–1970s. Their populations explosively expanded in the 1980s–1990s, and reduced some fish species to a threatened or endangered status [28]. In addition, similar to many other lakes in the world, Lake Biwa has experienced warming during the past several decades, which has also influenced the plankton community of the lake [24], [26]. Facing these ecosystem disturbances, the *G. isaza* population suddenly collapsed in the 1980s (Fig. 1c; data from [29]), although the detailed mechanisms remain unclear. Notably, this population decline dramatically increased the mean body size of survived fish through negative density dependency (Fig. 1b; data from [30]; also see [31]). At the same time, gammarids (*Jesogammarus annandalei*), another common prey of *G. isaza* [25], suddenly increased in population density through release from top-down regulation by *G. isaza* (Fig. 1d; also see [32]).

Long-term changes in the feeding habits of *G. isaza* have been the subject of previous research due to its unique characteristics and potential ecological importance. *G. isaza* is a freshwater goby endemic to Lake Biwa. *G. isaza* migrates from the pelagic to the littoral zone to breed during spring. The hatched larvae disperse offshore to grow from summer to winter, reaching maturity in the next spring [33]. They are usually annual and die after spawning, with some fish surviving to the second year. Intriguingly, *G. isaza* has adapted to a pelagic habitat with its strong swimming ability, whereas most gobiid fish are benthic. As a result of this strong swimming ability, this species plays important roles in the lake ecosystem as a keystone predator by feeding on pelagic zooplankton and benthic gammarid amphipods [25], [32], thereby coupling the pelagic and benthic food webs of the lake. Through stomach content analysis, Nakanishi and Nagoshi [25] reported that *G. isaza* fed mainly on zooplankton and gammarids in the 1960s. Their evaluation, however, was based on frequency of occurrence (i.e., presence/absence), which is a qualitative index that ignores numerical and weight composition of prey items in the diet. More recently, Ogawa et al. [34] and Nakazawa et al. [30] conducted stable isotope analysis of long-term specimens of *G. isaza* collected since the 1960s. Their stable isotope analyses provided useful information on the fish trophic dynamics. However, how the feeding habits of *G. isaza* have varied over the past decades remains unclear due to the lack of quantitative and direct evaluation of stomach contents.

In this study, our primary aim is to test the hypothesis that long-term variations in the diet composition of *G. isaza* would have been co-mediated by changes in both fish body size and environmental prey availability. We first provide >40-year time-series data of the diet composition of different body sizes of archived *G. isaza* specimens. We then investigate how the fish diet composition has been associated with temporal changes in environmental prey availability and fish body size by assessing prey selectivity, size-dependent feeding habits, and their combined effects. We also examine effects of eutrophication and fish body size on the diet composition. This additional analysis is motivated by the fact that long-term data on environmental prey availability are often unavailable in studies of fish feeding habits (unlike our case), and may help illustrate whether the use of lake trophic status data can be a good alternative in such cases.

## Materials and Methods

### Ethics statement

All scientific procedures were validated by the ethics committee of Center for Ecological Research, Kyoto University, and were conducted according to its guidelines and permits. Specimens of *G. isaza* collected from 1962 to 2004 during winter (mainly December) by commercial trawling (excluding 1991, 1992, and 1997 because of low abundance) were used for this study. The sampling scheme (i.e., location, depth, timing, and method) did not substantially change over time, and thus sampling bias is not a concern. Specimens were initially fixed in 10% formalin and subsequently preserved in 70% ethanol (see [34] for details). In the previous stable isotope study, Nakazawa et al. [30] analyzed 20 specimens per year selected in a manner representative of the body size range of each sampling year. For the present analysis, we used the same fish samples. The sample size might be small, yet it was because we cannot analyze many valuable historical specimens.

### Fish diet

We examined stomach contents of a total of 800 fish individuals (total body length ranging from 33 to 91 mm), among which 36 (4.5%) fish specimens had empty stomachs. We identified prey taxa and quantified these prey items per stomach by counting their undigested body parts under a compound microscope using a Sedgewick Rafter counting chamber. We found in this process that 17 (c.a. 2.1%) samples had only unidentifiable materials. These samples cannot be included in the following data analyses of diet composition, and we treated them as having empty stomachs for notational convenience. In addition, to estimate the prey biomass per stomach, we calculated the product of numerical count and average dry weight of each prey item. The average dry weight of each prey item was estimated by weighing all undigested individuals in bulk over the research period (after desiccation at 60°C for 24 hours) and dividing by the number of individuals. We adopted this approach because an exact evaluation of prey biomass was not possible due to digestion and mixture. Inter-annual variations of prey individual weight therefore could not be taken into account in the biomass evaluation; however, it does not matter to the major conclusions we draw. For small prey (*Bosmina* spp. and nauplii), length–weight relationships were used to estimate individual weight [35], because direct weight measurements were not possible. Although we recognize potential biases associated with our sampling and analytical methods or other factors such as prey-specific digestion rates and inter-annual variations of prey size, the present analyses represent the best quantitative data that can be obtained at present.

### Environmental prey availability and fish body size

The environmental prey data include annual average abundances of gammarids (*J. annandalei*) (Fig. 1d) from Center for Ecological Research, Kyoto University (see [32] for details), and zooplankton (*Daphnia* spp., *Bosmina* spp., *Leptodora kindtii*, Calanoida, and Cyclopoida) (Figs. 1e–i) from the Shiga Prefecture Fisheries Experimental Station (see [26] for details). These datasets are available for the period of 1966–2004 (excluding 2000) and 1962–2004 for gammarids and zooplankton, respectively. We used the annual average data because seasonal data were not completely available for all prey items. To examine effects of fish body size, we used the total body length data of the fish individuals used in the present stomach content analysis (see Fig. 1b; data from [30]).

### Data analysis

We evaluated temporal changes in three conventional indices of diet composition [36]: (i) frequency of occurrence (%F), which is the proportion of non-empty stomachs containing a particular prey item to all stomachs for each sampling year; (ii) numerical composition (%N), which is the proportion of the total numerical count of a particular prey item to total prey count among all fish stomachs in each year; and (iii) weight composition (%W), which is the proportion of biomass of a particular prey item to total prey biomass among all fish stomachs in each year. To analyze long-term trends of the diet composition, we calculated cumulative z-scores of the indices %N and %W for each prey item. The cumulative z-score analysis can be used to visually characterize the years with predominantly positive or negative anomalies and reveal the timing of trend initiation, although the timing cannot be tested statistically [17].

Using the diet composition data, we examined how the long-term variations of the fish feeding habits were affected by changes in environmental prey availability and fish body size. To carry out the data analyses, we focused on numerical rather than biomass data because feeding events occur at the individual level. Note also that most copepodites found in the diet were Calanoida, and thus, we aggregated Calanoida and copepodites into a single category of “Calanoida”. First, we compared the percentage of prey in the diet and in the environment (which hereafter referred to as “%prey in diet” and “%prey in environment,” respectively). In this analysis, we considered only the relative abundances (%N) of gammarids and zooplankton (*Daphnia* spp., *Bosmina* spp., *L. kindtii*, Calanoida, and Cyclopoida). We ignore other prey items (e.g., shrimps and juvenile fish) because no data are available on their environmental abundances; however, their overall contributions to the fish diet composition were small, and our conclusions would not be qualitatively changed. Note also that the gammarid abundance data were unavailable for 1962–1965 and 2000 (see above), and these five-year data could not be considered. Furthermore, fish samples with empty stomachs were also excluded because we are interested in the relative prey preference and diet composition; as a result, the sample size used in this analysis was *n* = 635. Thereafter, we also examined how the long-term variations of the feeding habits were affected by fish body size. Here, we used “prey numerical counts” of all prey items in the diet, so that all fish could be investigated including those with empty stomachs (i.e., *n* = 800). Note that the results were qualitatively the same when %prey in diet was used in this analysis.

To perform these two analyses, we used a quantile regression approach. This approach is used when ordinary least square regression analysis cannot be applied in the limiting cases [37]: for example, feeding selectivity of fish on different sizes of prey (e.g., zooplankton versus shrimps) depends critically on fish body size. That is, large fish may prefer large prey that typically have higher nutritious values, while small fish may feed only on small prey due to their gape limitation. As such, one may expect that the frequency of large prey items in the diet would increase with fish body size. However, this assumes that all large fish can find enough large prey for food. This assumption cannot be met if environmental availability of large prey is limited. When some large fish can meet sufficient number of large prey but others cannot (considering different years with different environmental conditions), a triangular distribution may occur, in which the upper limit of frequency of large prey items increases with fish body size, while the lower limit remains 0. A similar argument may be applied to small prey items as well. In performing the quantile regression analysis, we estimated the 0.01, 0.05, 0.25, 0.5, 0.75, 0.95, and 0.99 quantiles, following [38].

In addition to the univariate tests, we also investigated the combined effects of fish body size and %prey in environment on %prey in diet, using multiple regression analysis. Note again that in this analysis only the gammarid and zooplankton data were used as above (i.e., *n* = 635). These variables were normalized to unit mean and variance prior to analyses to show the relative contribution of each variable [39]. In order to avoid any specific parametric assumptions, we performed a randomization test (999 times) in assessing the significance of regression coefficients [40]. We did not carry out multivariate quantile regression, because its methodology and interpretation of the results are still under debate [38].

Finally, we examined how the individual and inter-annual variations of the diet composition were related to fish body size and eutrophication. We considered the annual average total phosphorus concentration (TP) in the upper 20 m of the water column (Fig. 1a) and total body length of fish individuals (see Fig. 1b) as possible determinants of %prey in diet. In this analysis, we considered all prey items (including minor ones) in all non-empty stomachs; as a result, *n* = 747. Here, we performed non-metric multi-dimensional scaling (NMDS). This is a coordination approach to visually summarize the variation of the data assemblages in a low dimensional space [41]. Bray-Curtis dissimilarity was used as the measure of pair-wise distances, and assemblage association was projected onto a two dimensional plot [41]. The NMDS scores of the first two axes were then linked to TP and fish body size using regression analyses. This approach is an indirect ordination analysis and avoids parametric assumption that might not be met for our data set. Finally, we also calculated the mean location (i.e., centroids) from the NMDS coordinates of all individual fish within a given year in order to visualize the inter-annual variations in diet composition.

## Results

### Overall diet composition

The diet of *G. isaza* consisted of various prey items (Table 1). A total of 5162 zooplankton, 1604 zoobenthos, and 51 unidentified juvenile fish were found in the diet (see Table 1 for details) When considering frequency of occurrence, gammarids (*J. annandalei*) were the most commonly found prey (%F = 66.3), followed by *Daphnia* (%F = 57.1). In terms of numerical composition, *Daphnia* was the most abundant prey (%N = 58.1) followed by gammarids (%N = 18.9%). In terms of weight composition, gammarids had the highest contribution (%W = 55.7) and *Daphnia* had the second highest contribution (%W = 13.0). The fish fed on many other prey such as *Bosmina* and copepods, chironomid larvae, juvenile fish, shrimps (*Palaemon paucidens*), and oligochaete worm, but their numerical and/or weight compositions were relatively small.

**Table 1:** Overall diet composition in terms of frequency of occurrence (%F), numerical composition (%N), and weight composition (%W).

Prey items	% F	% N	% W	Total counts	Average weight (mg)
Zooplankton					
*Bosmina* spp.	7.1	4.3	0.1	296	0.007(±0.016)
*Daphnia* spp.	57.1	58.1	13.0	3963	0.082(±0.742)
*Leptodora kindtii*	7.6	1.6	0.5	112	0.103(±0.165)
Calanoida	12.1	3.0	3.1	207	0.377(±0.816)
Cyclopoida	21.8	5.7	5.2	385	0.337(±1.091)
Copepodite	12.5	2.5	1.8	172	0.260(±0.156)
Nauplii	3.3	0.4	<0.01	27	0.0007(±0.0007)
Zoobenthos					
Oligochaete worm	3.6	1.3	0.4	86	0.111(±1.013)
Chironomid larvae	12.0	2.8	2.4	190	0.313(±0.761)
Gammarids	66.3	18.9	55.7	1291	1.075(±7.727)
Shrimps	4.6	0.5	10.3	37	6.954(±9.809)
Juvenile fish	6.0	0.8	7.6	51	3.700(±2.546)

The total individual counts and average individual dry weight (± standard deviation) during the entire research period is also listed for each prey item.

### Long-term changes in diet composition

**Figure 2:**
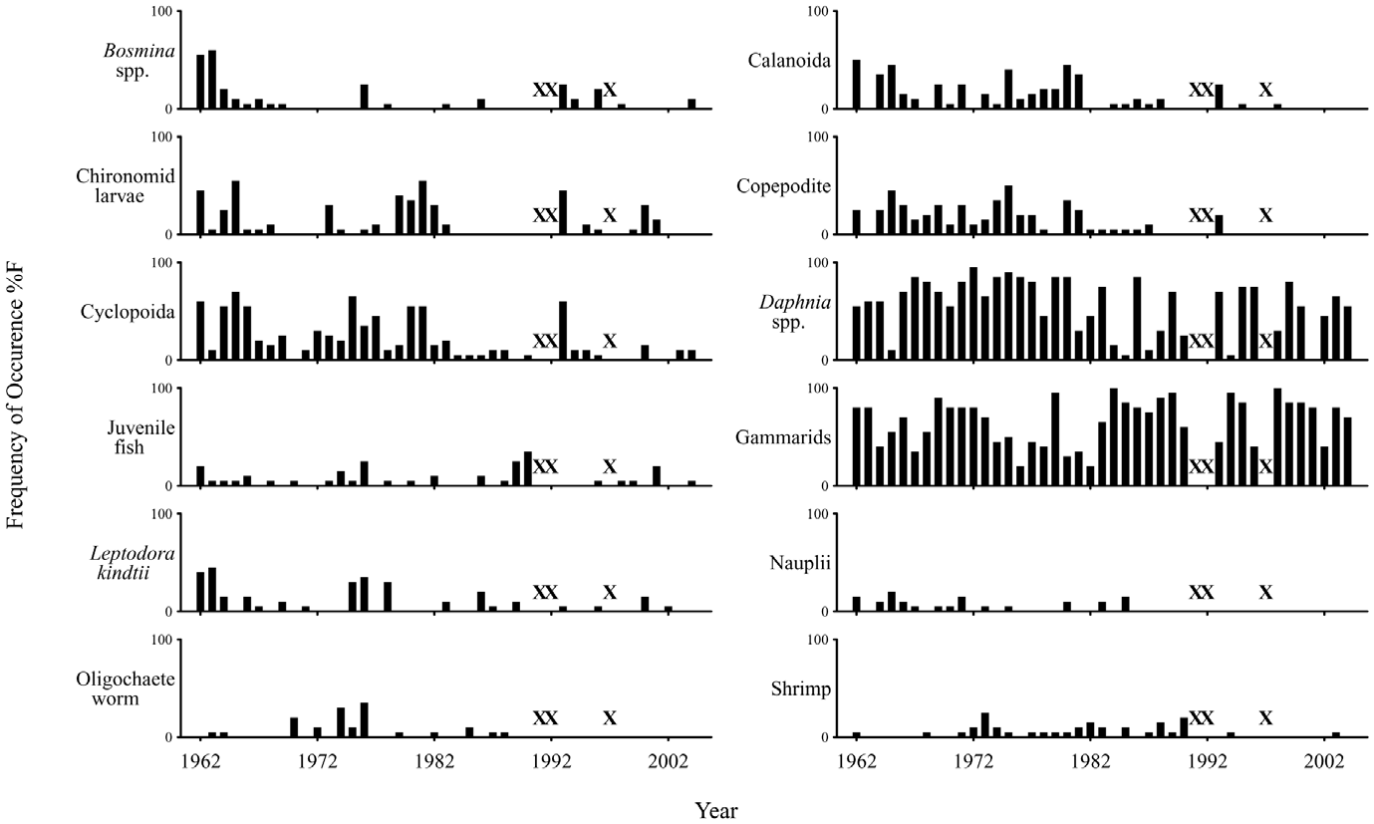
Time series of frequency of occurrence of each prey item in the diet. Crosses indicate years with no data.

**Figure 3:**
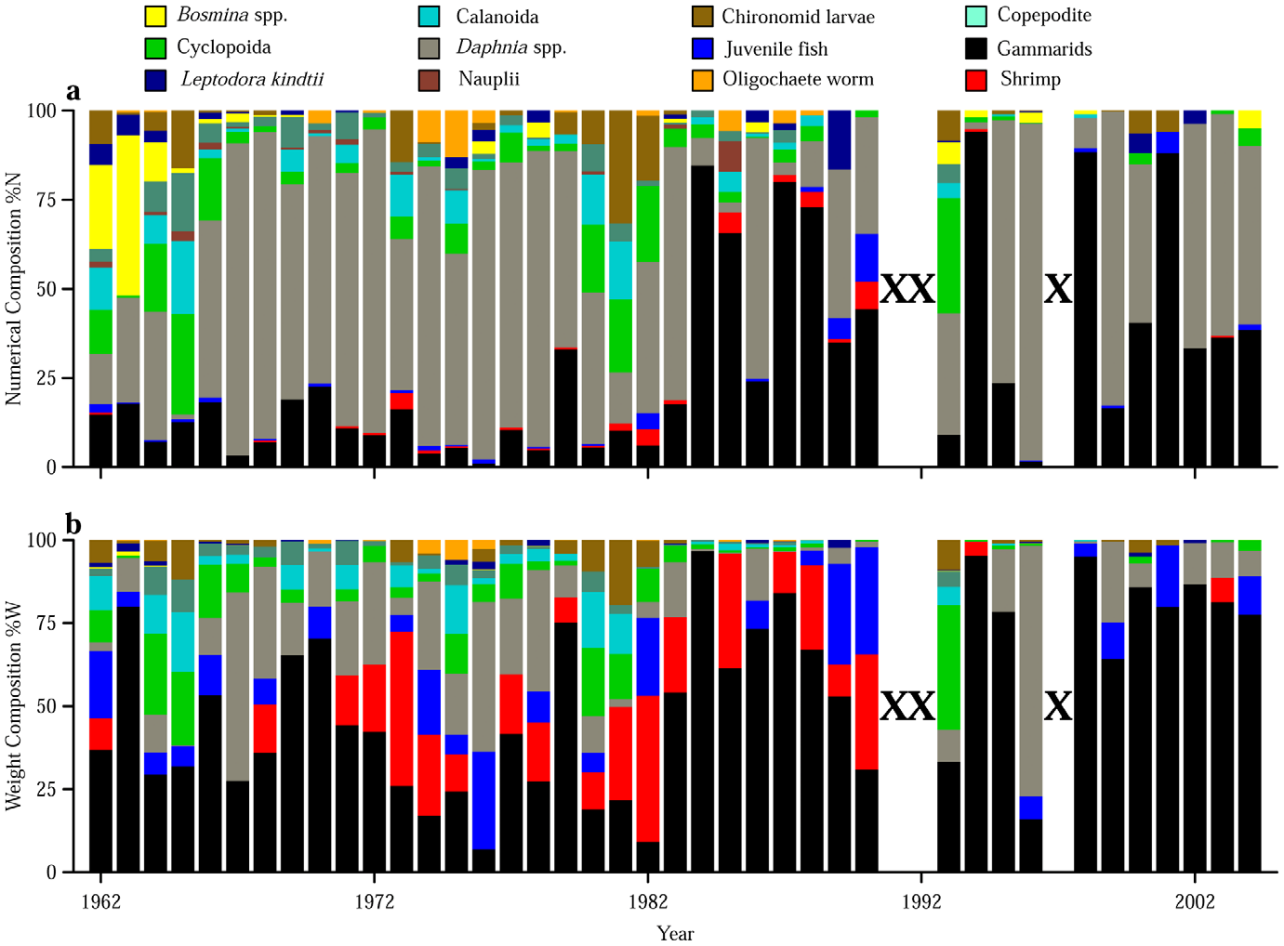
Time-series of composition of each prey item in the diet. (a) Numerical and (b) weight composition. Crosses indicate years with no data.

**Figure 4:**
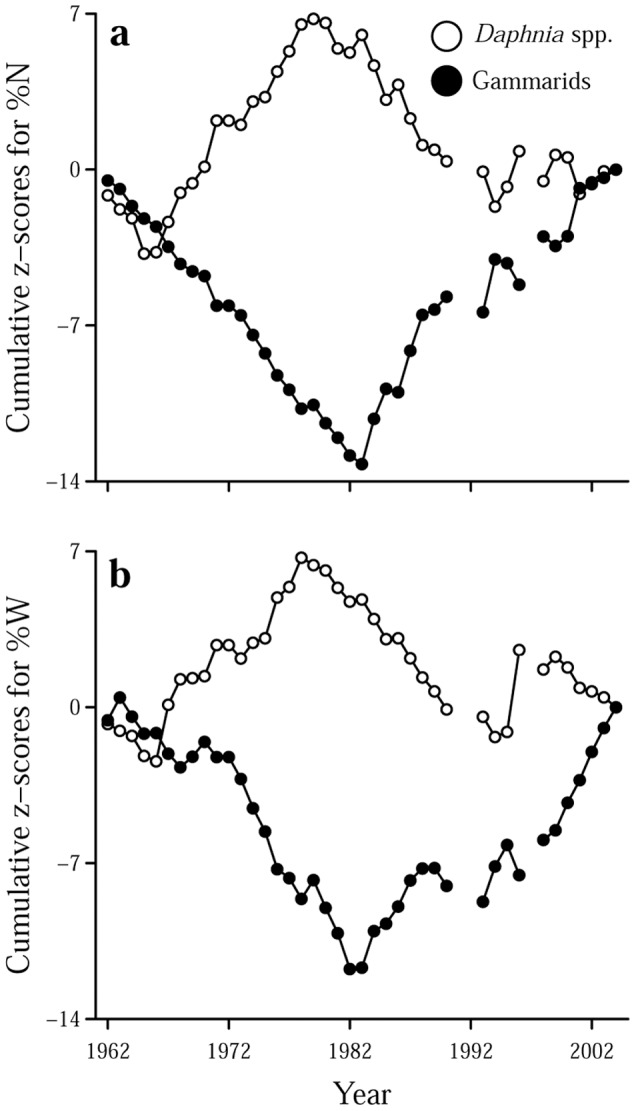
Cumulative z-score plots. (a) Numerical and (b) weight composition of *Daphnia* (open circles) and gammarids (closed circles).

Our time-series data showed a distinct diet shift starting in the 1980s. Frequency of occurrence (%F) showed that all prey items were commonly found in the diet during 1960s and 1970s, while many prey items decreased or were no longer found in the diet after the 1980s, except for *Daphnia* and gammarids that exhibited consistently high %F throughout the research period (Fig. 2). The results of the quantitative indices showed that zooplankton dominated the diet in terms of numerical composition (%N) from the 1960s to 1970s and *Daphnia* was especially prominent with %N ranging from 60 to 80% (Fig. 3a). The total weight composition (%W) of zooplankton prey was comparable to that of gammarids during the period (Fig. 3b), despite their having much lower individual weight than gammarids (Table 1). After the 1980s, %N of gammarids increased and was comparable to that of zooplankton (Fig. 3a), and gammarids began to strongly dominate the diet in terms of %W (60–80%) (Fig. 3b). Cumulative z-score plots revealed that %N and %W of *Daphnia* started to decrease in 1980 and 1983, respectively, and both %N and %W of gammarids started to increase in 1983 (Fig. 4).

### Effects of environmental prey availability and fish body size

**Figure 5:**
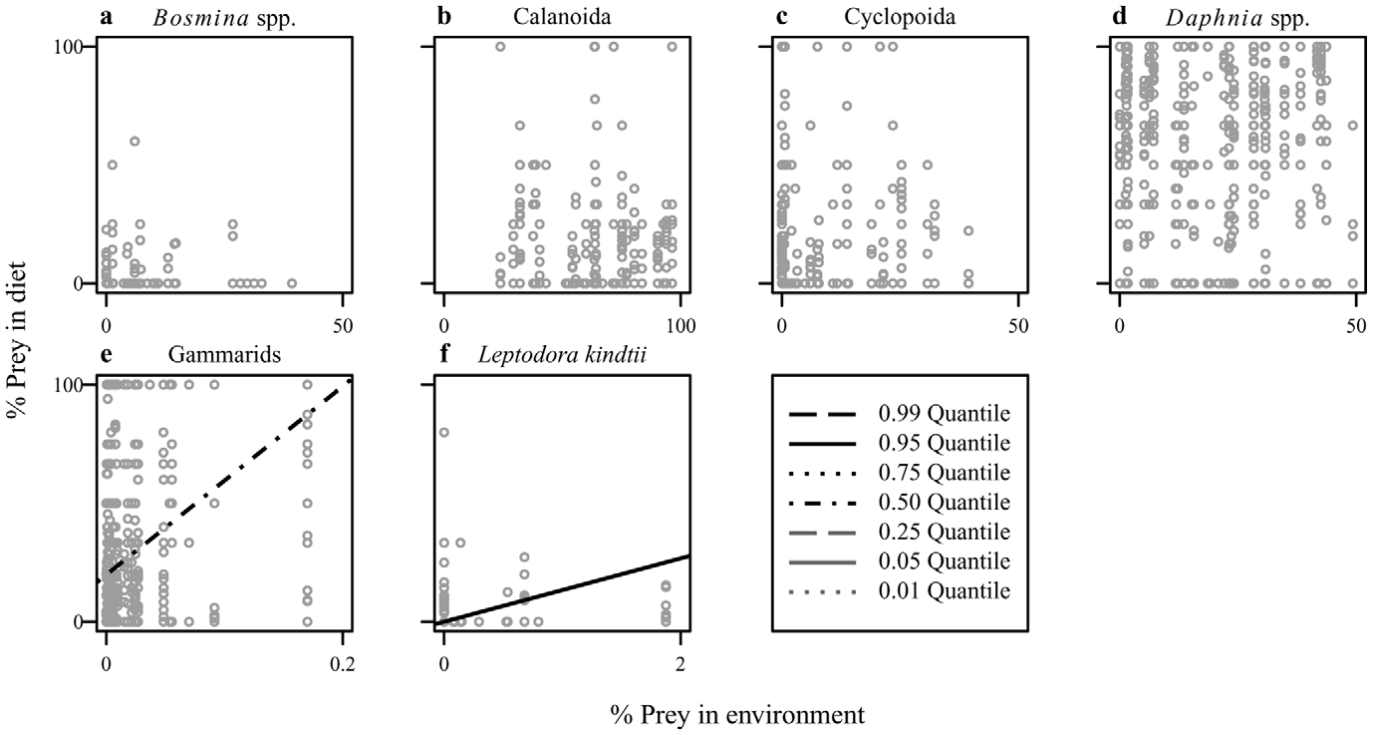
Scatter plot showing the relationships between numerical composition of prey in the diet and environment. (a) *Bosmina* spp., (b) Calanoida, (c) Cyclopoida, (d) *Daphnia* spp., (e) gammarids, and (f) *Leptodora kindtii*. Only significant (*p*<0.05) quantile regressions are shown.

**Figure 6:**
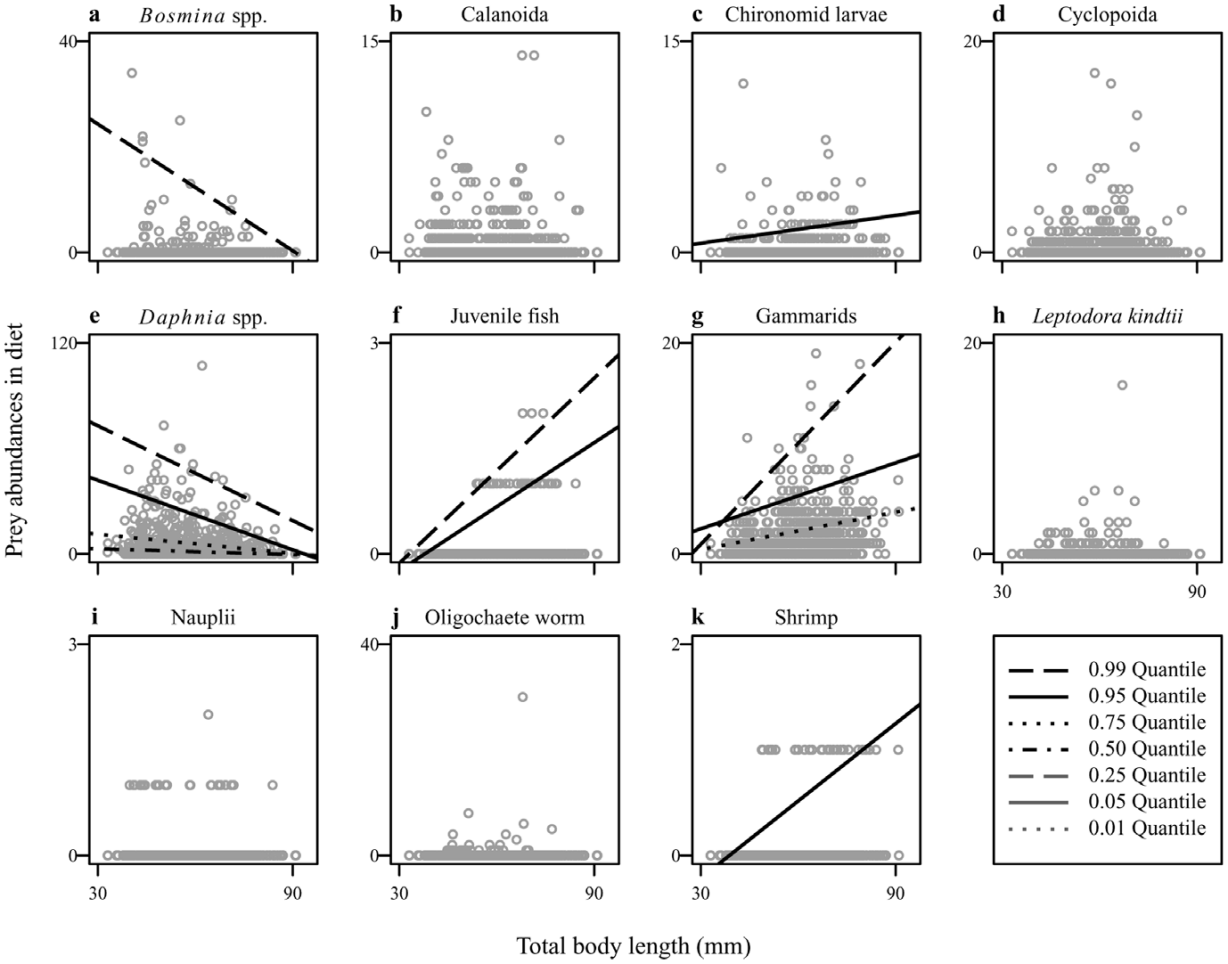
Scatter plot showing the relationships between prey abundance in the diet and fish body size. (a) *Bosmina* spp., (b) Calanoida, (c) chironomid larvae, (d) Cyclopoida, (e) *Daphnia* spp. (f) juvenile fish, (g) gammarids, (h) *Leptodora kindtii*, (i) nauplii, (j) Oligochaeta, and (k) shrimps. Only significant (*p*<0.05) quantile regressions are shown.

Quantile regression analyses showed that changes in the relative abundance of some prey items in the environment might have significant effects on the diet composition (Fig. 5). We found significant positive correlations between %prey in diet and environment for gammarids (Fig. 5e) and *L. kindtii* (Fig. 5f). No significant correlation was found for the other prey items (Figs. 5a–d). We also found that *G. isaza* exhibited size-dependent feeding habits (Fig. 6). Correlations between abundance in the diet and fish body size were significantly negative for *Bosmina* (Fig. 6a) and *Daphnia* (Fig. 6e) while positive for chironomid larvae (Fig. 6c), juvenile fish (Fig. 6f), gammarids (Fig. 6g), and shrimps (Fig. 6k). Thus, small and large fish individuals tended to feed on smaller (i.e., zooplankton) and larger (i.e., zoobenthos) prey items, respectively. Notably, these analyses showed that many prey items exhibited clear triangular distributions of dietary contribution in relation to fish body size. Thus, the upper limit of frequency of prey items changes with fish body size but the lower limit remains constant, which reflects a possible food limitation (see above).

Multiple regression analysis revealed relative effects of fish body size and environmental prey availability on the diet composition (Table 2). For *Daphnia*, %prey in diet was significantly negatively correlated with both fish body size and %prey in environment. For Calanoida, it was negatively correlated with fish body size. In contrast to these, significant positive correlations were found both between %prey in diet and environment and between %prey in diet and fish body size for gammarids. Importantly, the relative contribution of fish body size was larger than that of %prey in environment for both *Daphnia* and gammarids. The interaction term was not significant for any of the prey items investigated.

**Table 2:** Results of multiple regression analysis on the relationships between diet composition versus fish body size and environmental prey availability.

Prey items	*β*_1_	*β*_2_	*β*_3_
*Bosmina* spp.	NS	NS	NS
*Daphnia* spp.	−0.264**	−0.077*	NS
*Leptodora kindtii*	NS	NS	NS
Cyclopoida	NS	NS	NS
Calanoida	−0.122**	NS	NS
Gammarids	0.304**	0.168**	NS

The full model is tested in the form: (prey composition in gut) = *β*_1_×(fish body size)+*β*_2_×(relative prey abundance in environment)+*β*_3_×(fish body size)×(relative prey abundance in environment). A randomization test was performed 999 times.

* : *p*<0.05,

** : *p*<0.01, NS: non-significance (*p*>0.05).

**Figure 7:**
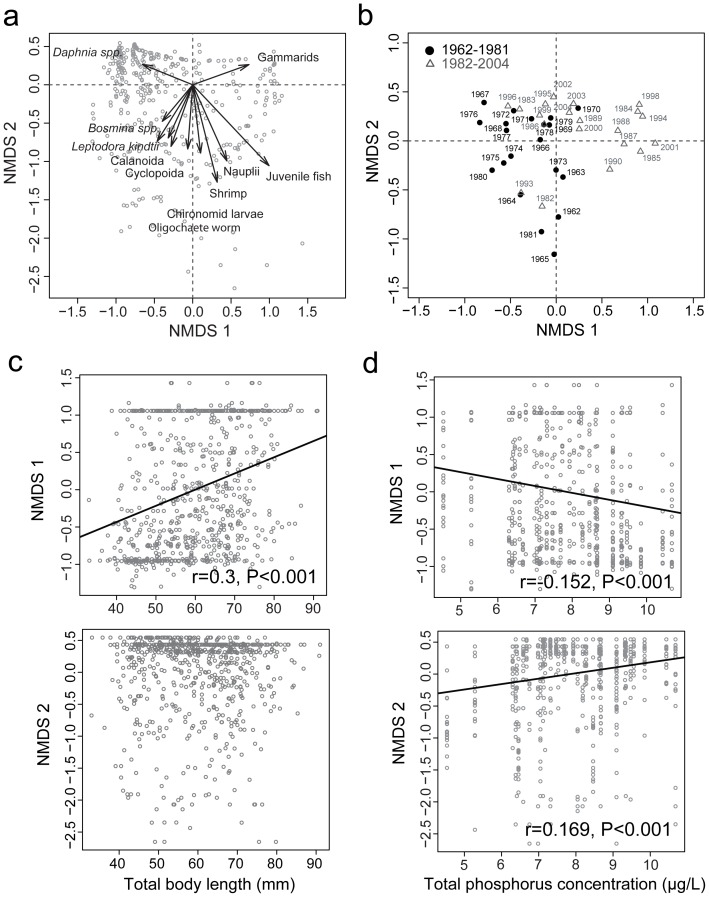
Non-metric multi-dimensional scaling (NMDS) of the diet composition in relation to fish body size and total phosphorous concentration (TP). (a) NMDS biplot summarizes variations of the diet composition among individual fish. Dots represent individual data points, and arrows indicate score vectors for corresponding prey items. (b) The annual means (centroids) of individual NMDS coordinates. Closed circles and open triangles are for the periods of 1962–1982 and 1983–2004, respectively. Correlations are tested between NMDS scores versus (c) fish body size and (d) TP.

NMDS analysis summarized variations of the diet composition among individual fish in relation to fish body size and total phosphorus concentration (TP) (Fig. 7). Obviously, the composition of small (i.e., zooplankton) and large (i.e., zoobenthos and juvenile fish) prey in the diet were associated positively and negatively with the first (NMDS1) axis, respectively (Fig. 7a). This is especially true for *Daphnia* and gammarids, although not always for the other minor prey items. In contrast, the second (NMDS2) axis is strongly negatively related to the minor prey items (Fig. 7a). The annual mean scores were clearly separated on the NMDS1 and NDMS2 axes into two groups for the periods before and after the diet shift in 1982 (Fig. 7b). We found a significant positive correlation between NMDS1 score and fish body size (*r* = 0.3, *p*<0.001; Fig. 7c), while NMDS1 and NMDS2 scores were negatively (*r* = −0.152, *p*<0.001) and positively (*r* = 0.169, *p*<0.001) correlated with TP, respectively (Fig. 7d).

## Discussion

We performed a detailed analysis of stomach contents of *G. isaza* collected over the past 40 years. The fish fed on various prey items, among which zooplankton (especially *Daphnia*) and gammarids were the major items (Table 1; Figs. 2 and 3). This is consistent with previous qualitative dietary analysis based on frequency of occurrence during the 1960s [25]. Nevertheless, with our updated quantitative analyses on the fish from 1962 to 2004, we found that the numerical and weight composition of prey items in the diet have substantially changed through time, shifting from zooplankton to predominantly gammarids in the 1980s (Figs. 2–4).

Our diet analyses supported the hypothesis that the long-term variations in the diet composition of *G. isaza* were linked to inter-annual fluctuations of both environmental prey availability and fish body size. Although this is not surprising in light of previous research, such long-term evidence has rarely been available especially in freshwater ecosystems. We also emphasize that we obtained qualitatively new insights into the fish feeding habits co-mediated by changes in fish body size and environmental prey availability. We found positive effects of environmental prey availability only for gammarids (Fig. 5; Table 2), which suggests that *G. isaza* has a general preference for gammarids over zooplankton prey. Counterintuitively, a negative relationship was found between %prey in diet and environment for *Daphnia* and Calanoid in the multiple regression analysis (Table 2). This may simply result from an apparent negative correlation between the proportions of zooplankton and gammarids in the diet. We observed (Fig. 6; Table 2) that small fish fed mainly on small prey (mainly *Daphnia*), likely due to gape limitation, while large fish preferred large prey (mainly gammarids), likely because larger prey would be more nutritious. Intriguingly, the effects of fish body size on the diet composition were stronger than those of environmental prey availability. This is illustrated by the result of multiple regression analyses in the cases where the effects of these two factors are both significant (i.e., *Daphnia* and gammarids) (Table 2).

These results provide the following scenario for the observed changes in the feeding habits of *G. isaza*. Until the 1980s, zooplankton availability was high relative to that of gammarids (Figs. 1d–i; also see [26], [32]) and body size of *G. isaza* was relatively small (Fig. 1b; also see [31]). These conditions would have forced the fish to utilize zooplankton (small prey) as the main food item. After the 1980s, however, gammarid availability increased relative to zooplankton and fish body size also increased as a result of the fish population collapse [31], [32]. These changes would have allowed the fish to shift to gammarids as the predominant prey. This scenario can be supported by the optimal foraging theory, which predicts that predators feed only (or mainly) on the most profitable prey based on maximizing energy gain per handling time [42], [43]. If we follow this theory, we can infer that gammarids were the most profitable prey for *G. isaza* after the 1980s owing to reduced handling time (i.e., increased fish body size; Fig. 1b) and larger per-capita nutrition (i.e., body size) than zooplankton (Table 1). Such a size-based view of foraging behavior is useful for a better understanding of food-web impacts of fish predation and is increasingly recognized as important in the analysis of food-web structure [8], [44]. The present findings are significant in that they could separate the confounding effects of body size and prey availability on the fish feeding habits, and provide good evidence that, as well as population/community dynamics, body size dynamics are also crucial for long-term changes in fish feeding habits and thus aquatic food webs (see also below).

The effects of fish body size and total phosphorus concentration (as a proxy for environmental prey availability) were also significant in the NMDS analysis (Fig. 7). In particular, the positive correlation between NMDS1 and fish body size can support that larger and smaller fish prefer gammarids and *Daphnia*, respectively (compare Figs. 7a and 7c). On the other hand, the NMDS2 axis seemed to capture the minor prey composition in the diet (Fig. 7a), and its negative correlation with phosphorus concentration (Fig. 7d) may reflect the observation that the minor prey almost disappeared in the diet after the 1980s following the alleviation of eutrophication (Figs. 2–3). We also found a positive correlation between NMDS1 and phosphorus concentration (Fig. 7d), which would be a spurious correlation due to the temporal coincidence of the alleviation of eutrophication and increases in fish body size in the 1980s (Fig. 1). These multivariate results further corroborate our conclusions based on the comparison with environmental prey availability. However, some of these explanations remain speculative and caution should be exercised. This is probably because apparent negative correlations exist among the proportions of prey items in the diet (see also above) and/or because environmental changes influence the diet composition indirectly via complex prey responses (e.g., competition and predation within the prey community).

In the present study, we showed that *G. isaza* had consumed both zooplankton (mainly *Daphnia*) and zoobenthos (mainly gammarids) as its major prey and its relative reliance had varied over time in relation to changes in fish body size and environmental prey availability (Figs. 2–4). In Lake Biwa, gammarids (*J. annandalei*) comprise the largest biomass among the benthos community [45], and its population dynamics are largely determined by top-down regulation of *G. isaza* [32]. Considering these situations, our data have significant implications in the pelagic-benthic coupling in the lake. The role of the pelagic-benthic coupling in lake food-web structure and dynamics has been identified recently and is gaining increasing attention [3], [46], [47]. However, their research focus has been on how pelagic-benthic coupling by omnivorous fish predation mediates food-web responses to environmental changes [48], [49]. Therefore, most studies have not considered fish body size as a major determinant of pelagic-benthic coupling (but see [18], [19] for within-year variations due to ontogenetic niche shifts). Indeed, food-web models for pelagic-benthic coupling have assumed that the omnivorous fish has a species-specific (i.e., size-independent) prey preference [50], [51] (also see [52]). Notably, the present study suggests that in order to better understand the functional roles of pelagic-benthic coupling in lake food-web dynamics, not only prey availability but also fish body size should be considered. These arguments are generally applicable, because zooplankton and zoobenthos are distinct in body size and also because fish body size and prey availability are both likely to fluctuate over time in lake ecosystems [22].

Finally, we briefly discuss that stomach content analysis has some advantages over stable isotope analysis especially in the long-term study of fish feeding habits, although both approaches have advantages and disadvantages that make them complimentary and should be used in concert [20]. In previous studies, Ogawa et al. [34] and Nakazawa et al. [30] examined long-term variations in nitrogen stable isotope ratios (*δ* ^15^N) of archival specimens of *G. isaza*. Their data showed that *δ* ^15^N of the fish was drastically enriched in the 1960s and 1970s during the eutrophication period. Ogawa et al. [34] further showed that the isotope signature was synchronized with a proxy of pelagic primary products extracted from the chronological sediment core samples. Then, they concluded that the fish trophic level had remained almost constant through the latter half of the 20th century. In the present study, however, we showed that the diet composition of the fish drastically changed around the 1980s (Figs. 2–4). This apparent inconsistency can be explained by the fact that in Lake Biwa, *Daphnia* and gammarids, the main prey items of *G. isaza*, are basically primary consumers feeding on phytoplankton or its sinking debris, respectively, and they have similar isotope signatures [53], [54]. As a result, the observed diet shift from *Daphnia* to gammarids in the 1980s is detectable only by direct stomach content analysis (see Fig. S1 in File S1 for preliminary comparison between estimated *δ* ^15^N of fish individuals based on stomach contents versus direct stable isotope measurements of the fish). We should be more aware of the fact that, while stable isotope studies can provide useful information on fish trophic dynamics, they alone may not be able to tell us much about temporal changes in interspecific interactions within a food web (e.g., pelagic-benthic coupling; see above).

In conclusion, we reported long-term changes in the diet composition of *G. isaza*, and our detailed analysis of the diet data revealed how the long-term variations in the diet composition would have been co-mediated by changes in both fish body size and environmental prey availability. These results provide the best long-term evidence in freshwater ecosystems that fish feeding habits vary over decades with changes in its body size and prey community structure due to anthropogenic disturbances such as eutrophication (oligotrophication) and fish population collapse. We emphasize that detailed time-series of size-associated diet data, such as ours, are laborious to collect, yet quite important for more completely understanding aquatic food webs responding to ecosystem disturbances.

## Supporting Information

File S1 (DOC)
