# Supplementary material for: Long-Term Changes in the Diet of Gymnogobius isaza from Lake Biwa, Japan: Effects of Body Size and Environmental Prey Availability
Source: PLoS One. 2012 Dec 28;7(12):e53167. doi: 10.1371/journal.pone.0053167 (PMC3532214; doi:10.1371/journal.pone.0053167)
Supplement: File S1 — (DOC) [file pone.0053167.s001.doc]

**Comparison between stomach content and stable isotope data**

First, we calculated expected nitrogen stable isotope ratios (*δ*^15^N) of fish individuals based on measured *δ*^15^N of prey items and their weight composition (%W) in the fish diet. Assuming a constant fractionation coefficient of 3.4‰ per trophic level (Minagawa and Wada 1984), we defined that (expected *δ*^15^N of a fish individual) = 3.4‰ + Σ(*δ*^15^N of prey item *i*) × (the fraction of prey item *i* in the diet). Here we considered only fish individuals having non-empty stomachs; thus, *n* = 747. We used the following values for prey *δ*^15^N: 11.6‰ (*Daphnia* spp.), 15.0‰ (*Leptodora kindtii*), 13.8‰ (Calanoida), 15.9‰ (Cyclopoida), 13.8‰ (copepodite), 11.02‰ (oligochaete worm), 12.2‰ (chironomid larvae), 11.4‰(gammarids), 12.55‰ (shrimps), and 13.85‰ (juvenile fish). These data are from Kiyashko et al. (2001) for chironomid larvae and from Sakai (unpublished data) for the other prey items. Note that *δ*^15^N data are not available for copepodite, *Bosmina* spp., and nauplii. For copepodite, we used Calanoida *δ*^15^N because they may be considered to have similar feeding habits. For *Bosmina* spp. and nauplii, we ignored them because their %W was minor (Table 1), thereby excluding two fish specimens from the analysis. We plotted expected *δ*^15^N of fish individuals over the research period (top panel of Fig. S1).

Then, we compared expected and measured *δ*^15^N of the fish. The measured *δ*^15^N data are from Nakazawa et al. (2010). Ideally, expected *δ*^15^N of the fish estimated from stomach contents should agree with that of direct stable isotope measurements (i.e., a positive correlation with a slope = 1). However, the regression analysis showed that the correlation between expected and measured *δ*^15^N values was weakly negative (slope = -0.100, *n* = 745, *p* < 0.05; lower panel of Fig. S1). In general, such an inconsistency may suggest methodological differences between stomach content and stable isotope analyses (i.e., time integrated versus snapshot information on diet). However, another and more important reason here would be that, because no data were available on temporal (i.e., inter-annual and seasonal) changes in prey *δ*^15^N, we needed to assume that prey *δ*^15^N was constant over the 40 years. Using sediment core samples, Ogawa et al. (2001) previously showed that *δ*^15^N of pelagic primary products would be drastically enriched in the 1960s and 1970s during the eutrophication period in Lake Biwa. This implies that prey *δ*^15^N would be lower (i.e., we overestimated fish *δ*^15^N) for the earlier research period. We also note that species-specific *δ*^15^N data of zooplankton and zoobenthos were still limited and we could not yet fully consider prey isotopic variations. Therefore, the present analysis is still preliminary and the simplified assumptions made here should be revisited for more detailed analyses in future work that aims to investigate long-term trophic dynamics of the fish. That could give more convincing arguments about the comparison between stomach content and stable isotope results.

**References**

1. Kiyashko SI, Narita, T, Wada, E (2001) Contribution of methanotrophs to freshwater macroinvertebrates: evidence from stable isotope ratios. Aquat Microb Ecol24: 203–207.
2. Minagawa M, Wada E (1984) Stepwise enrichment of *δ*^15^N along food chains: further evidence and the relation between *δ*^15^N and animal age. Geochim Cosmochim Acta 48: 1135–1140.
3. Nakazawa T, Sakai Y, Hsieh CH, Koitabashi T, Tayasu I, et al. (2010) Is the relationship between body-size and trophic niche position time-invariant in a predatory fish? First stable isotope evidence. PLoS ONE 5: e9120.
4. Ogawa N, Koitabashi T, Nakamura T, Oda H, Ohkouchi N, et al. (2001) Fluctuations of nitrogen isotope ratio of gobiid fish (Isaza) specimens and sediments in Lake Biwa, Japan, during the 20th century. Limnol Oceanogr 46: 1228–1236.

**Figure S1**
